# Evaluation of Modern Approaches for the Assessment of Dietary Carotenoids as Markers for Fruit and Vegetable Consumption

**DOI:** 10.3390/nu15071665

**Published:** 2023-03-29

**Authors:** Thorsten Henning, Philipp Wagner, Egbert Gedat, Bastian Kochlik, Paula Kusch, Kay Sowoidnich, Marko Vastag, Jeannine Gleim, Marcel Braune, Martin Maiwald, Bernd Sumpf, Tilman Grune, Daniela Weber

**Affiliations:** 1 Department of Molecular Toxicology, German Institute of Human Nutrition Potsdam-Rehbruecke (DIfE), 14558 Nuthetal, Germany; thorsten.henning@dife.de (T.H.); bastian.kochlik@dife.de (B.K.); paula.kusch@dife.de (P.K.); scientific.director@dife.de (T.G.); 2 Food4Future (F4F), c/o Leibniz Institute of Vegetable and Ornamental Crops (IGZ), 14979 Grossbeeren, Germany; 3 Institute of Nutritional Science, University of Potsdam, 14469 Potsdam, Germany; 4 Faculty of Engineering and Natural Sciences, Wildau Technical University of Applied Sciences, 15745 Wildau, Germany; wagner@th-wildau.de (P.W.); gedat@th-wildau.de (E.G.); 5 NutriAct Competence Cluster Nutrition Research Berlin-Potsdam, 14558 Nuthetal, Germany; 6 Ferdinand-Braun-Institut (FBH), Leibniz-Institut für Höchstfrequenztechnik, 12489 Berlin, Germany; kay.sowoidnich@fbh-berlin.de (K.S.); martin.maiwald@fbh-berlin.de (M.M.); bernd.sumpf@fbh-berlin.de (B.S.); 7 Department of Physiological Chemistry, Faculty of Chemistry, University of Vienna, 1090 Vienna, Austria

**Keywords:** carotenoids, vitamins, e-health, app, dietary record, blood, skin measurements, spectroscopy, Raman

## Abstract

The assessment of dietary carotenoids via blood measurements has been widely used as a marker for fruit and vegetable consumption. In the present study, modern, non-invasive approaches to assess dietary carotenoids, such as skin measurements and an app-based short dietary record (ASDR), were compared with conventional methods such as plasma status and handwritten 3-day dietary records. In an 8-week observational study, 21 healthy participants aged 50–65 years recorded their daily consumption of carotenoid-rich fruits and vegetables via a specially developed ASDR. Anthropometry, blood samplings and assessment of skin carotenoids via Raman and reflection spectroscopy were performed at baseline, after four weeks and at the end of the study. App-based intake data showed good correlations with plasma α-carotene (r = 0.74, *p* < 0.0001), β-carotene (r = 0.71, *p* < 0.0001), and total plasma carotenoids (r = 0.65, *p* < 0.0001); weak correlations with plasma lutein/zeaxanthin and β-cryptoxanthin (both r = 0.34, *p* < 0.05); and no correlation with plasma lycopene. Skin measurements via reflection and Raman spectroscopy correlated well with total plasma carotenoids (r = 0.81 and 0.72, respectively; both *p* < 0.0001), α-carotene (r = 0.75–0.62, *p* < 0.0001), and β-carotene (r = 0.79–0.71, *p* < 0.0001); moderately with plasma lutein/zeaxanthin (both r = 0.51, *p* < 0.0001); weakly with plasma β-cryptoxanthin (r = 0.40–0.31, *p* < 0.05); and showed no correlation with plasma lycopene. Skin measurements could provide a more convenient and noninvasive approach of estimating a person’s fruit and vegetable consumption compared to traditional methods, especially in studies that do not intend blood sampling. ASDR records might function as a suitable, convenient tool for dietary assessment in nutritional intervention studies.

## 1. Introduction

A healthy diet including sufficient consumption of fruits and vegetables is associated with a reduced risk for chronic noncommunicable diseases such as type 2 diabetes, hypertension, macular degeneration, and colorectal cancer [1,2,3,4]. In 2017, eleven million deaths worldwide were attributed to dietary risk factors, of which low fruit consumption alone accounted for two million deaths [5]. The World Health Organization’s (WHO) recommendation for a healthy diet includes the daily consumption of a minimum of 400 g of various fruits and vegetables; however, this value is only reached by few countries [6]. Beside their high content in fiber and minerals, fruits and vegetables are a good source for antioxidative phytochemicals such as carotenoids. Carotenoids belong to a group of fat-soluble pigments, found in high concentrations especially in red, yellow, and dark green, leafy vegetables. The most important carotenoids in human nutrition are α- and β-carotene, lycopene, lutein, zeaxanthin and β-cryptoxanthin [7]. The assessment of circulating plasma and serum carotenoids has been widely accepted as a suitable marker for fruit and vegetable consumption [8,9,10]. Due to their antioxidative and anti-inflammatory properties, a diet high in carotenoids has been linked to reduce risks for chronic diseases including type 2 diabetes, macular degeneration and several types of cancers [11,12,13,14], diseases which are also known to be mediated by F/V consumption. Thus, the precise assessment of dietary carotenoids could play a vital role for the early prevention of such diseases, especially in vulnerable groups [15]. Assessment of carotenoids is usually carried out by HPLC coupled with UV or mass spectrometric detection. Although HPLC analysis is considered the gold standard [16], its disadvantages such as elaborate sample handling and analysis or inconvenient blood collections drives research to find novel approaches that add up to traditional assessment tools.

In addition to conventional blood analyses and dietary protocols, spectroscopic skin measurements are increasingly used for the detection of carotenoid in human nutrition [17]. Due to their photoprotective as well as skin coloring properties, carotenoids have gained increasing interest leading to a rise in carotenoid supplements intended for endogenous skin protection and tanning usually containing β-carotene, lycopene, astaxanthin, lutein or mixtures of those carotenoids [7,18]. Upon intake, carotenoids accumulate in lipophilic compartments such as subcutaneous adipose tissue, but can also be found throughout the dermis and epidermis [7]. Spectroscopic methods, such as reflection or Raman spectroscopy, offer the possibility to non-invasively detect carotenoids in skin layers, possibly replacing blood analyses [19]. Miniaturizations of the measuring systems over the years to tabletop devices have made the use of spectroscopic approaches for self-tracking of carotenoid status increasingly interesting in large-scale epidemiological studies.

Classical dietary assessment methods such as food frequency questionnaires (FFQ) or 3-day dietary records to estimate carotenoid intake are often laborious and time-consuming, and are susceptible to errors due to delayed or forgotten entries. Digital approaches of monitoring dietary intake, especially via smart phone applications (short: app), enable the user to track their food consumption quickly and instantaneously. This allows study personnel, especially in research settings, to monitor and check the completeness of participants’ datasets in real time and thus to intervene if necessary.

The aim of this study was to evaluate the potential of spectroscopic skin measurements to replace blood tests for carotenoid analysis as well as the eligibility of an app-based short dietary record (ASDR) for carotenoid intake compared to conventional dietary assessment tools.

## 2. Materials and Methods

Participants in this study included men and women aged between 50 and 65 years and with an BMI between 19 and 29 kg/m^2^ from the Potsdam area, Germany. Twenty-one healthy participants — including thirteen females and eight males — were enrolled at the Human Study Center of the German Institute of Human Nutrition in Potsdam-Rehbruecke, Germany. Participants were considered for the study if they were confident with using a smartphone and available Wi-Fi to transfer data via the study app. Active smoking (≥4 cigarettes/day), diabetes, and cancer as well chronic diseases of the intestine, liver, kidney, and pancreas were considered as exclusion criteria. Enrollment was conducted from June to September 2022. The study duration for participants was eight weeks including three visits at the study center at baseline, after four weeks and after eight weeks. Throughout the study period, participants recorded their daily consumption of carotenoid-rich food items using an ASDR. During the visits, anthropometric measurements (height, weight, bioimpedance analysis and grip strength), an assessment of skin carotenoids (via Raman and reflection spectroscopy), as well as a short questionnaire to evaluate the study app (last visit) were performed. Additionally, throughout the 8-week study, two 3-day dietary records (classical DR) had to be completed. Fasting EDTA plasma (4.9 mL) was collected on each visit and stored at −80 °C until further analyses.

### 2.1. Plasma Biomarkers

The carotenoids lutein/zeaxanthin, β-cryptoxanthin, lycopene and α-, β-carotene, as well as the vitamins α-, γ-tocopherol and retinol, were simultaneously analyzed in plasma using high-performance liquid chromatography (HPLC) with UV and fluorescence detection, as previously described [20]. In brief, 30 µL of plasma was extracted with 150 µL of extraction solution (*n*-butanol/ethanol, 1:1, *v*/*v*) containing β-apo-8′-carotenal-methyloxime as an internal standard. After vigorous mixing and centrifugation, the supernatant was transferred into autosampler vials. A total of 20 µL were injected into the HPLC system (Shimadzu Prominence LC-20A, Duisburg, Germany) and detected via UV (all carotenoids and the internal standard) and fluorescence (retinol and tocopherols), respectively. Quantification was performed with pure standard mixtures verified against Standard Reference Material (SRM 968f, NIST, Gaithersburg, MD, USA). Plasma cholesterol was analyzed using an enzymatic method, described by Deeg et al. [21] using a Cobas Mira autoanalyzer (Roche, Mannheim, Germany). Plasma triglycerides were determined by the CHOD-PAP method using the Cobas Mira autoanalyzer (Roche, Mannheim, Germany).

### 2.2. Skin Carotenoid Assessment

Raman spectroscopy: A prototype measurement system based on shifted excitation resonance Raman difference spectroscopy (SERRDS), as described here [22], was used for the assessment of skin carotenoids. Measurements were performed on the palm of the hand in the hypothenar region using a dual-wavelength diode laser as excitation light source. A spot diameter of 3 mm and a power density of 1270 W/m^2^ on the skin were chosen to comply with legal regulations for maximum permissible exposure levels for skin. The excitation wavelengths were λ_1_ = 487.2 and λ_2_ = 487.6 nm. As the excitation wavelengths fall into a spectral region where carotenoids absorb strongly, the intensity of characteristic carotenoid Raman signals is enhanced by up to 6 orders of magnitude and will, thus, be well above the Raman signal intensities of other skin components (e.g., proteins or lipids). In this way, a selective and sensitive detection of carotenoids in skin can be accomplished. However, the chosen excitation wavelength also induces strong fluorescence interference. To separate the Raman signals from such interfering fluorescence backgrounds a difference spectrum from the two generated Raman spectra is calculated. This derivative-shaped difference spectrum is then converted into a Raman spectrum in conventional form by means of numerical integration. For the specific detection of skin carotenoids, a Raman band resulting from a C=C stretching vibration at a wavenumber of approx. 1525 cm^−1^ was used as this repesented the most intense carotenoid Raman signal in the investigated spectral range. The Raman signal height was used as a measure for the carotenoid content. Measurements were performed in triplicate and the means of those measurements were used for analysis. To account for potential fluctuations in laser power, an external intensity calibration was performed daily using polystyrene and intensities were normalized according to the reference standard.

Reflection spectroscopy: Skin carotenoids were assessed using pressure-mediated reflection spectroscopy with a commercially available reflection spectroscope (Veggie Meter^®^, Longevity Link Corporation, Salt Lake City, UT, USA) equipped with a white LED source exciting the skin carotenoids in the spectral range of 350–850 nm and detecting their diffusely reflected light. Measurements were performed as an average of three scans on the index fingers of both hands, whereas the mean of both hands was used for statistical analysis. The resulting carotenoid reflection score (CRS) was expressed as an arbitrary unit on a scale from 0 to 900. Prior to each measurement, the Veggie Meter^®^ was calibrated using a dark and white reference blank. Furthermore, participants were asked to wash and dry their hands and use hand sanitizer prior the measurements. After each use, the lense of the Veggie Meter^®^ was cleaned with an optical cloth as suggested by the manufacturer [23].

### 2.3. Assessment of Dietary Carotenoids

**Figure 1:**
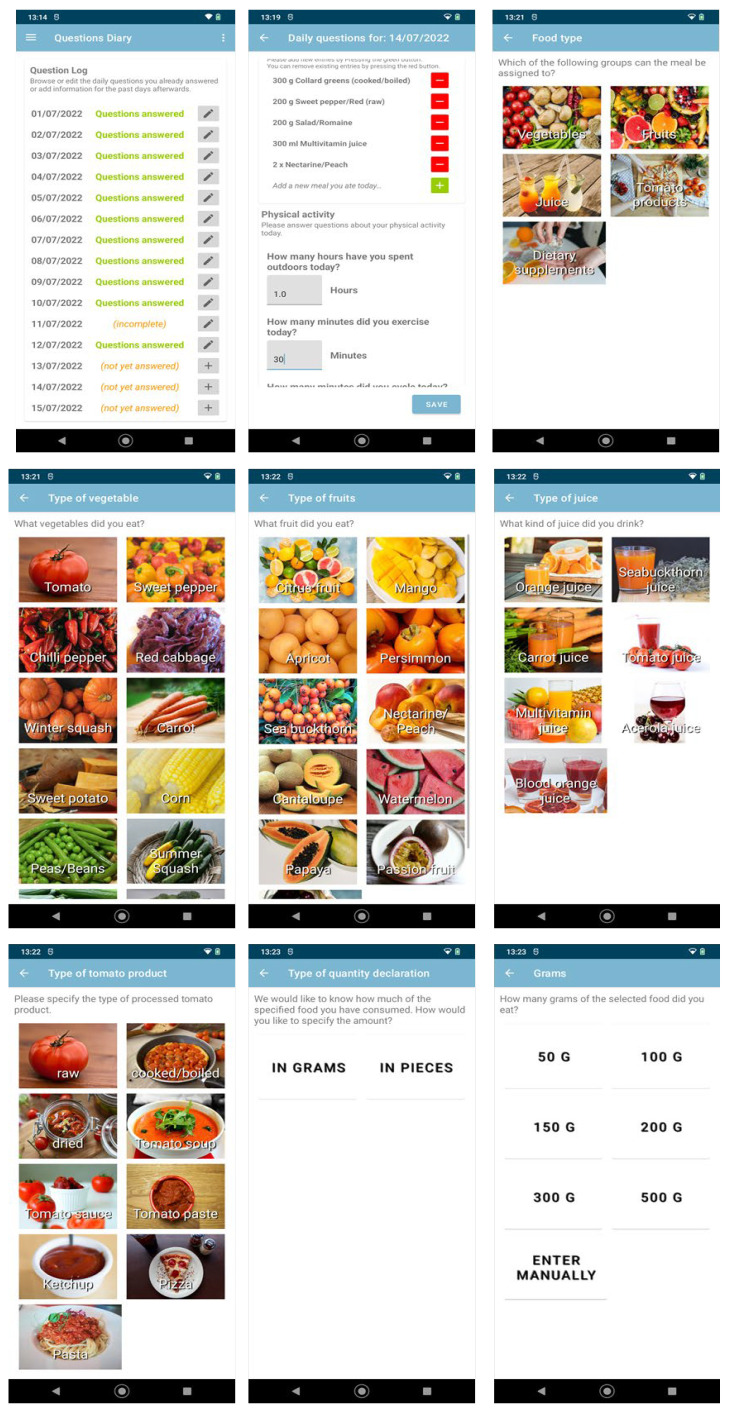
Study app used for daily tracking of carotenoid-rich food items.

ASDR: Participants were asked to track their daily dietary sources of carotenoid-rich foodstuffs, including fruits, vegetables and juices using a specially developed study app (Figure 1). Subjects were asked to indicate the amount of fruits and vegetables consumed, in grams (respectively, in ml for juices) or as a number of pieces, as well as the processing status (vegetables: raw, cooked, or canned; fruits: raw, dried, or canned). A complete list of the food items included in the app can be found in Supplemental List S1. To adequately record lycopene intake, participants were additionally asked to track the amount of processed tomato products (e.g., tomato paste, ketchup, soup, pizza, and pasta). For the specific recording of dietary supplements, a bar code scanner was included in the app. The daily intake of all dietary carotenoids (mg/d) was calculated using data from the USDA (U.S. Department of Agriculture) Standard Reference Legacy database (released April 2018) [24]. Reference weights for fruits and vegetables were used from the German Federal Office of Consumer Protection and Food Safety [25]. The app was developed for the Android platform and is compatible with the Android Versions 9 to 13. Participants were allowed to use their own Android smartphones or were provided with a separate device if their mobile smartphone ran a different operating system (e.g., iOS or Windows) or did not want to install the study app on their private phone. The participants’ food consumption input was initially cached on the device and periodically transferred in the background via internet to a backend server operated by the IT center of the Wildau Technical University of Applied Sciences. The backend server ran a service application developed specifically for use in this study, which provided a RESTful (representational state transfer) API (application programming interface) endpoint. This allowed a structured end-to-end-encrypted communication with the app, managed authentication and authorization for participants, study personnel and scientists, and managed and encapsulated all database access. The backend service application additionally provides the smartphone app with the study-relevant content to be displayed to the participants such as selectable foods, images and categories. The consumption data itself is stored in the schema-less DBMS (Database Management System) MongoDB (MongoDB Inc., New York City, NY, USA, Version 4.4.13), which is only accessible through the backend service application. For further processing, the acquired data was then retrieved by connecting to the same backend server authenticating as a user with a scientist role who received read access to all pseudonymized collected data. The initial processing and analysis of the data collected by the app was performed using MATLAB (The Mathworks Inc., Nattick, MA, USA, Version 9.11.0 (R2021b)). For comparison with skin and plasma carotenoids, four-week mean values of ASDR-based carotenoid intake were calculated for each participant.

3-Day dietary record: Within the first four study weeks, subjects completed a 3-day dietary record and again within the second four study weeks. Participants were asked to select representative days for their dietary protocols, with at least one weekend day included. The evaluation of the dietary records was performed by trained nutritionists using PRODI^®^ 6.5 software (Nutri-Science GmbH, Freiburg, Germany). PRODI^®^ is an organizational software for nutritional counseling and nutritional therapy widely used in nutritional research, practices, and clinics in Germany. For the calculation of daily β-carotene intake (mg/d), PRODI^®^ utilizes the German Nutrient Data Base (Bundeslebensmittelschlüssel; BLS) [26]. As values for lycopene, α-carotene and lutein/zeaxanthin and β-cryptoxanthin were missing for most food items in the BLS, β-carotene intake retrieved from the app data was only compared to β-carotene intake assessed by 3-day dietary records.

### 2.4. Statistical Analysis

The normal distribution of variables was analyzed using the Shapiro-Wilk test. If the data were not normally distributed, the data were transformed to achieve normal distribution by log or square root tranformation. Tranformed variables are presented as geometric mean with 95% confidence intervall (CI) and untransformed values are presented as means ± standard deviation (SD). Pairwise correlations between different methods for carotenoid assessment were performed using Pearson correlation with transformed data. Correlations were regarded as strong for Pearson correlation coefficients r ≥ 0.80, good for 0.79 ≥ r ≥ 0.60, moderate for 0.59 ≥ r ≥ 0.40 and, weak for r < 0.40. Differences between timepoints (baseline, week 4, and week 8) were assessed using repeated measures ANOVA or paired-samples *t*-test, respectively. All statistical analyses were carried out using SPSS software (SPSS Inc., Chicago, IL, USA; Version 25).

## 3. Results

### 3.1. Participants’ Characteristics

Twenty-two participants came in for baseline testing, with one participant feeling unable to use a smartphone, who was, therefore, excluded. A total of 21 participants completed the study. The study cohort was mostly female (72%) with a mean age of 57.7 ± 4.9 years, a mean BMI of 23.6 ± 3.1 kg/m^2^, and a mean waist-to-hip ratio of 0.85 ± 0.09. The average fat mass was 21.0 ± 6.4 kg while the mean skeletal muscle mass was 22.1 ± 6.3 kg. The detailed baseline characteristics of the study population are shown in Table 1.

The plasma levels of α-carotene and β-carotene significantly increased over the whole study period between baseline, week 4, and week 8, while plasma levels of α-tocopherol and total carotenoids significantly increased between baseline and week 4 (Table 2). Skin levels of carotenoids assessed with reflection spectroscopy significantly increased between baseline and week 8, whereas no significant increase in carotenoid skin levels measured with Raman spectroscopy was observed. With regard to other fat-soluble micronutrients and their intake data, no significant differences over the study period were observed (see Table 2).

### 3.2. Skin Measurements

**Figure 2:**
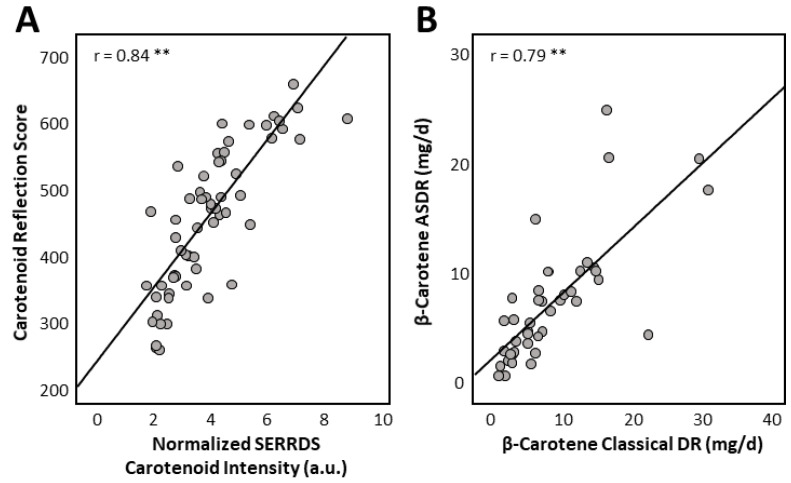
(**A**) Correlation between skin carotenoid measurements performed with Raman and reflection spectroscopy from 21 subjects assessed at three visits (*n* = 63). (**B**) Correlation between intake-data for β-carotene assessed using app-based short dietary record and classical 3-day dietary record (*n* = 42). Displayed Pearson correlation coefficients were calculated from transformed data. ** *p* < 0.0001.

Skin levels assessed by reflection spectroscopy (carotenoid reflection score; CRS) showed a strong correlation with skin levels measured with Raman spectroscopy (SERRDS) (r = 0.84, *p* < 0.0001, Figure 2). The CRS correlated strongly with total plasma carotenoids (r = 0.81, *p* < 0.0001), while the SERRDS carotenoid intensity (CI) showed good correlation with total plasma carotenoids (r = 0.72, *p* < 0.0001) (see Table 3).

Regarding single-carotenoid plasma levels, α- and β-carotene correlated best with carotenoid reflection scores (CRS) and SERRDS CI (see Table 3), whereas the plasma levels of lutein/zeaxanthin moderately correlated with both skin measurements. The contribution of β-cryptoxanthin to skin levels was only weak, and for lycopene, no significant correlation with skin levels was observed.

### 3.3. Dietary Intake Data

The daily dietary intake of β-carotene assessed using the 3-day dietary record showed good correlations with the corresponding app-based intake data (r = 0.79, *p* < 0.0001; Figure 2). The app data correlated well with the plasma levels of α-carotene, β-carotene and total carotenoids, whereas the correlations between intake data on β-cryptoxanthin and lutein/zeaxanthin with plasma levels were weak (see Table 3). No correlation between the intake data and plasma lycopene levels was found. The dietary intake data for single carotenoids showed overall moderate correlations with skin levels assessed using Raman and reflection spectroscopy (see Table 4), with the exception of lycopene which was not associated with either skin measurement. ASDR data of β-cryptoxanthin only showed weak correlations with skin levels using reflection spectroscopy.

## 4. Discussion

The current study aimed to explore the suitability of modern, non-invasive methods for carotenoid assessment versus established methods. Good-to-strong correlations between skin carotenoid status measured with reflection and Raman spectroscopy and total carotenoids in plasma were found, which are in line with correlations found in previous studies ranging from r = 0.68 to 0.77 [27,28,29,30,31]. Regarding single carotenoids, skin levels showed the highest correlations with plasma levels of β-carotene, α-carotene and lutein/zeaxanthin, whereas plasma β-cryptoxanthin only correlated weakly with skin levels measured with reflection spectroscopy. Lycopene showed no correlation with skin levels independent of the measuring system (Table 3). The results are in good agreement with data from Matsumoto et al., who assessed skin and serum carotenoids in 811 Japanese men and women [30]. In that study, total plasma carotenoids (r = 0.68), β-carotene (r = 0.65) and α-carotene (r = 0.62) showed good correlations with skin carotenoid levels, whereas plasma lutein (r = 0.41) and β-cryptoxanthin (r = 0.48) showed weaker correlations with skin levels. Additionally, plasma lycopene showed the weakest correlation (r = 0.28) with skin values beside zeaxanthin (r = 0.16) which was, unlike in the present study, not recorded as a sum parameter together with lutein [30]. One reason why no correlation between the plasma levels of lycopene and SERRDS carotenoid intensity was found in the study may be related to the excitation wavelength of the prototype Raman instrument. In this study, the excitation wavelengths of 487.2 and 487.6 nm were used for measuring of skin carotenoids, whereas for an isolated measurement of lycopene, a wavelength of approximately 515 nm was described as more suitable [32]. Consequently, an improved Raman system employing excitation wavelengths around 488 nm and 515 nm could provide additional insights regarding the specific detection of different carotenoids. In the present work, only the intensity of the strongest carotenoid Raman signal around 1525 cm^−1^ was considered for data analysis. It is expected that the incorporation of further Raman signal parameters, e.g., signal width or signal area, or the analysis of an extended spectral range containing further carotenoid Raman signals, has the potential to improve correlations with the classic blood analysis. The aforementioned study also reported a slow response in skin lycopene levels compared to plasma levels after undergoing a lycopene-deprived diet followed by a daily supplementation with 25 mg of lycopene [32]. Jahns et al. observed a strong increase in both skin carotenoid levels measured with Raman spectroscopy (λ = 488 nm) and total plasma carotenoids following an eight-week daily intake of approximately 62 mg dietary carotenoids, including daily consumption of lycopene-rich food items. Thus, the increase in skin carotenoids was not attributed to individual carotenoids in that study [27].

Regarding correlations between retrospective dietary assessment methods including FFQs and 24 h dietary recalls, low correlations are reported in the literature. While Morgan et al. found no associations between total plasma carotenoids and self-reported fruit and vegetable intake assessed by a 19-item FFQ and web-based 24 h dietary recalls carried out on multiple days [29], other studies reported positive but weak correlations between self-reported fruits and vegetables dietary intake and total plasma carotenoid data ranging from r = 0.24–0.36 [15,28,30]. The current study revealed good correlations between dietary intake data assessed with an ASDR and total carotenoids (r = 0.65), β-carotene (r = 0.71) and α-carotene (r = 0.74) with their corresponding plasma levels while only weak to no correlations were found regarding lutein/zeaxanthin, β-cryptoxanthin and lycopene. These findings are in line with findings from Fraser et al., who reported stronger correlations between dietary intake data for α- and β-carotene (r = 0.49–0.62) with their corresponding plasma levels, compared to lutein/zeaxanthin, lycopene and β-cryptoxanthin in 909 subjects from the Adventist Health Study 2 [9]. Low correlations between dietary lycopene and their corresponding plasma levels could possibly be explained by individual in vivo isomerisation rates. While dietary lycopene is mainly taken up in its natural *all-trans* form [33], *cis*-isomers make up more than 50% of total lycopene in human plasma and tissues [34,35]. The molar absorption coefficients for *cis*-lycopenes vary greatly in dependency of their structure being ε = 60.076 for 13-*cis* lycopene, ε = 117.199 for 9-*cis* lycopene and ε = 183.717 L/mol·cm [36]. In the present study, the chromatographic separation of *trans*-lycopene from its *cis*-isomers was not performed. This could have led to the underestimation of plasma lycopene levels especially in participants with individually high *cis*-lycopene levels.

Plasma values of α-, β- and total carotenoids showed a stronger association with dietary intake assessed with the ASDR than skin measurements, whereas skin measurements showed a stronger association with dietary lutein/zeaxanthin intake than plasma values (see Table 3 and Table 4). Moreover, skin levels assessed with Raman spectroscopy were a moderate predictor for β-cryptoxanthin intake. This observation might be explained by the different absorption rates of each carotenoid. The bioavailability of the xanthophylls lutein and β-cryptoxanthin is described to be higher compared to carotenes such as α- and β-carotene [37,38]. Upon intake, lutein and β-cryptoxanthin might reach their target tissues faster than carotenes. Similarly, the clearance of xanthophylls in plasma might occur faster compared to carotenes, explaining the lower correlations between dietary intake and plasma concentrations for β-cryptoxanthin and lutein/zeaxanthin. The combined accumulation time of total carotenoids in the skin upon increased intake is described to be in the range of 1–2 weeks [27,39].

The overall dietary intake of total carotenoids in the study group (12.5–15.1 mg/d) assessed with the ASDR was comparable to intake data described in other observational studies, ranging from 10.8 to 16.9 mg/d [9,28,29]. The average intake of carotenoids was not statistically significantly different between the first four and the last four study weeks. A reason for this might be due to a diminishing behavioural effect of the daily app usage on the participants’ fruit and vegetable consumption. Thus, when being asked whether the daily tracking of carotenoid-rich food items had an impact on their daily fruit and vegetable consumption, 38.1% of the study participants reported that the daily use of the app increased their fruit and vegetable consumption compared to their intake prior to the study (see Supplemental Table S1). Thus, 61.9% reported no change in their dietary behaviour.

Limitations of the present study include the small study population which did not allow in-depth subgroup analysis. As PRODI^®^ uses the German Nutrient Database for the nutritional evaluation of food items, intake data from the ASDR were only compared to β-carotene intake data from the 3-day dietary records since carotenoid values for α-carotene, lutein/zeaxanthin, β-cryptoxanthin and lycopene are missing in the German Nutrient Database. As the ASDR only covers carotenoid-rich food items, it does not provide information about the daily intake of, e.g., proteins, fat, carbohydrates or the energy of the users. However, the app framework was designed to be quickly adapted to changing requirements or different research questions. The strength of the current study is a detailed and comprehensive monitoring of study participants’ carotenoid consumption using both conventional and novel assessment methods.

## 5. Conclusions

In the current study, the usability of an app-based short dietary record for tracking the consumption of carotenoid-rich food items against 3-day dietary records, skin carotenoid scores, and plasma carotenoid levels was demonstrated. Non-invasive assessment methods for dietary carotenoids including ASDR and skin measurements via Raman and reflection spectroscopy were significantly associated with blood status of carotenoids, especially for α-carotene, β-carotene, and total carotenoids. Modern approaches for the assessment of dietary carotenoids as markers for fruit and vegetable consumption might offer an adequate alternative for blood assessment, especially in research settings where blood draws are not intended. App-based short dietary records might function as a suitable, convenient tool for dietary assessment in nutritional intervention studies.

## Figures and Tables

**Table 1:** Participant Baseline Characteristics (*n* = 21).

**Characteristic**	
Age (years)	57.7 ± 4.9
Sex, male %, (*n*)	38.1 (8)
Height (cm)	170.2 ± 9.7
Weight (kg)	68.7 ± 13.0
BMI (kg/m^2^)	23.6 ± 3.1
Waist-to-hip ratio	0.85 ± 0.09
Fat mass (kg)	21.0 ± 6.4
Relative fat mass (%)	30.7 ± 7.3
Skeletal muscle mass (kg)	22.1 ± 6.3
Fat free mass (kg)	47.9 ± 11.1
Grip strength (kg)	33.8 ± 10.3
Daily exercise (min)	23.4 ± 19.7
Average steps/day	7204 ± 3267
Supplement use, % (*n*)	42.9 (9)
***Time requirement for App usage (%)***	
<5 min	38.1
5–10 min	33.3
>10 min	28.6

Values are given as mean ± SD or percentage (*n*).

**Table 2:** Plasma concentrations, skin levels and dietary intake data of fat-soluble micronutrients over the study period.

	Baseline	Week 4	Week 8	*p*-Value
***Plasma***				
Total Carotenoids (µM)	3.68 (2.98–4.55) ^a^	4.35 (3.59–5.27) ^a,b^	4.59 (3.71–5.67) ^b^	0.013
α-Carotene (µM)	0.35 (0.21–0.61) ^a^	0.52 (0.32–0.84) ^b^	0.61 (0.38–0.96) ^c^	0.002
β-Carotene (µM)	1.28 (0.94–1.75) ^a^	1.62 (1.22–2.15) ^b^	1.72 (1.26–2.35) ^c^	0.012
Lutein/Zeaxanthin (µM)	0.55 (0.48–0.63)	0.61 (0.53–0.70)	0.63 (0.53–0.75)	0.097
Lycopene (µM)	0.68 (0.59–0.80)	0.75 (0.66–0.85)	0.72 (0.61–0.86)	0.521
β-Cryptoxanthin (µM)	0.47 (0.37–0.61)	0.51 (0.43–0.62)	0.50 (0.42–0.60)	0.461
Retinol (µM)	2.03 ± 0.51	2.10 ± 0.51	2.06 ± 0.50	0.432
γ-Tocopherol (µM)	1.19 ± 0.77	1.22 ± 0.62	1.19 ± 0.45	0.974
α-Tocopherol (µM)	32.5 ± 6.4 ^a^	35.7 ± 6.8 ^b^	34.2 ± 6.1 ^a,b^	0.024
Cholesterol (mM)	5.59 ± 0.80	5.70 ± 0.77	5.76 ± 0.94	0.156
Triglycerides (mM)	1.03 ± 0.40	1.00 ± 0.46	1.00 ± 0.36	0.748
***Skin Carotenoid levels***				
CRS (a.u.)	419 (371–470) ^a^	441 (391–494) ^a,b^	472 (423–523) ^b^	0.005
SERRDS CI (a.u.)	3.74 (3.16–4.44)	3.54 (2.92–4.28)	3.95 (3.28–4.75)	0.370
		**Week 1–4**	**Week 5–8**	***p*** **-Value**
***App-based short dietary record***				
Total Carotenoids (mg/d)	-	15.1 (13.0–17.3)	12.5 (10.2–15.2)	0.124
α-Carotene (mg/d)	-	1.40 (0.83–2.35)	1.00 (0.43–2.32)	0.322
β-Carotene (mg/d)	-	5.56 (3.89–7.95)	4.46 (2.71–7.34)	0.187
Lutein/Zeaxanthin (mg/d)	-	1.44 (0.94–2.21)	1.20 (0.80–1.81)	0.186
Lycopene (mg/d)	-	4.23 (2.91–6.15)	4.13 (2.77–6.16)	0.496
β-Cryptoxanthin (mg/d)	-	0.18 (0.11–0.24)	0.20 (0.13–0.24)	0.713
***3-Day dietary record***				
β-Carotene (mg/d)	-	6.80 (4.71–9.80)	5.64 (3.78–8.40)	0.198

Values are given as geometric mean (95% CI) or mean ± SD. Differences between groups were calculated by repeated-measure ANOVA or paired samples *t*-test. Bonferroni post hoc test was used for assessment of individual group differences. Values sharing a common superscript letter are not significantly different. a.u. = arbitrary unit. Significance considered at *p* < 0.05.

**Table 3:** Correlations between specific plasma carotenoids from week 4 and week 8 with their respective skin levels from week 4 and week 8 and intake data from weeks 1–4 and weeks 5–8 (*n* = 42).

Plasma	CRS	SERRDS CI	ASDR
Total Carotenoids	0.81 **	0.72 **	0.65 **
α-Carotene	0.75 **	0.62 **	0.74 **
β-Carotene	0.79 **	0.71 **	0.71 **
Lutein/Zeaxanthin	0.51 **	0.51 **	0.34 *
Lycopene	0.25	0.13	0.27
β-Cryptoxanthin	0.40 *	0.31	0.34 *

Pairwise correlation between carotenoid assessment methods were calculated using Pearson correlation. * *p* < 0.05, ** *p* < 0.0001.

**Table 4:** Correlations between single dietary carotenoids assessed using app-based short dietary record in weeks 1–4 and weeks 5–8 with skin levels from week 4 and week 8 (*n* = 42).

ASDR	CRS	SERRDS CI
Total Carotenoids	0.53 **	0.53 **
α-Carotene	0.52 **	0.48 *
β-Carotene	0.58 **	0.53 **
Lutein/Zeaxanthin	0.54 **	0.46 *
Lycopene	0.14	0.13
β-Cryptoxanthin	0.38 *	0.57 **

Pairwise correlation between carotenoid assessment methods were calculated using Pearson correlation. Significance * *p* < 0.05, ** *p* < 0.0001.

## Data Availability

The data that support the findings of this study are available from the corresponding author upon reasonable written request.

## Supplementary Materials

The following supporting information can be downloaded at: https://www.mdpi.com/article/10.3390/nu15071665/s1, File S1: List of foodstuffs included in the App-based short dietary record; Table S1: Behavioral effects of daily app-usage.
