# Supplementary material for: Evaluation of Modern Approaches for the Assessment of Dietary Carotenoids as Markers for Fruit and Vegetable Consumption
Source: Nutrients. 2023 Mar 29;15(7):1665. doi: 10.3390/nu15071665 (PMC10097221; doi:10.3390/nu15071665)
Supplement: Supplementary file 1 [file nutrients-15-01665-s001.zip › Supplementary File S1_Food_Items.pdf]

Food list App-based short dietary record

Vegetables:

Tomatoes, red, ripe, raw, year-round average
Tomatoes, red, ripe, cooked
Tomatoes, red, ripe, canned,
Tomatoes, sun-dried
Peppers, sweet, red, raw
Peppers, sweet, red, cooked,
Peppers, sweet, yellow, raw
Peppers, sweet, yellow, cooked
Peppers, sweet, green, raw
Peppers, sweet, green, cooked
Peppers, hot chili, red, raw
Carrots, raw
Carrots, cooked
Carrots, canned
Sweet potato, cooked, baked in skin
Pumpkin, raw
Pumpkin, cooked
Pumpkin, canned
Corn, sweet, yellow, raw
Corn, sweet, yellow, cooked
Corn, sweet, yellow, canned
Peas, green, raw
Peas, green, cooked
Peas, green, canned
Cucumber, peeled, raw
Pickles, cucumber, sour
Broccoli, raw
Broccoli, cooked
Arugula, raw
Lettuce, iceberg
Lettuce, cos or romaine, raw
Lettuce, butterhead, raw
Lettuce, red leaf (lollo rosso), raw
Lettuce, green leaf, raw
Lettuce, endivie, raw
Spinach, raw
Spinach, cooked
Kale, raw
Kale, cooked
Collards, raw
Collards, cooked
Cabbage, red, raw
Cabbage, red, cooked, boiled, drained, without salt

Fruits:

Oranges, raw, all commercial varieties
Tangerines, (mandarin oranges), raw
Grapefruit, raw, pink and red
Mangos, raw
Mango, dried, sweetened
Apricots, raw
Apricots, dried,
Melons, cantaloupe, raw
Watermelon, raw
Papayas, raw
Nectarines, raw
Cherries, sour, red, raw
Passion-fruit, (granadilla), purple, raw
Persimmons, raw
Sea buckthorn, raw

Juices

Orange juice, canned, unsweetened
Tomato juice, canned,
Carrot juice, canned
Multivitamin juice (mainly known and consumed in Germany)
Acerola juice, raw
Sea buckthorn juice

Processed tomato products

Tomato soup, condensed
Tomato, canned, sauce
Tomato, canned, paste
Catsup
Pizza
Pasta with tomato sauce
