# Supplementary material for: Evaluation of Modern Approaches for the Assessment of Dietary Carotenoids as Markers for Fruit and Vegetable Consumption
Source: Nutrients. 2023 Mar 29;15(7):1665. doi: 10.3390/nu15071665 (PMC10097221; doi:10.3390/nu15071665)
Supplement: Supplementary file 1 [file nutrients-15-01665-s001.zip › Supplementary Table S1_App_Evaluation.pdf]

Supplemental Table S1: Behavioural effects of daily-app usage (n=21)

Did the App-usage have an influence on your daily…	*Yes, increased*	*Yes, decreased*	*No impact*
…F/V consumption, % (n)	38.1 (10)	0 (0)	61.9 (12)
…Stress level, % (n)	19.0 (4)	4.8 (1)	76.2 (16)
…Physical activity, % (n)	14.3 (3)	0 (0)	85.7 (18)
